# A statistical approach to automated analysis of the low‐contrast object detectability test for the large ACR MRI phantom

**DOI:** 10.1002/acm2.70173

**Published:** 2025-07-14

**Authors:** Ali M. Golestani, Julia M. Gee

**Affiliations:** ^1^ Department of Physics and Astronomy University of Calgary Calgary Alberta Canada; ^2^ Department of Medical Physics Alberta Health Services Calgary Alberta Canada; ^3^ Department of Oncology University of Calgary Calgary Alberta Canada; ^4^ College of Engineering and Physical Sciences University of Guelph Guelph Ontario Canada

**Keywords:** large ACR Phantom, low‐contrast object detectability, MRI, Python code, quality control (QC)

## Abstract

**Background:**

Regular quality control checks are essential to ensure the quality of MRI systems. The American College of Radiology (ACR) has developed a standardized large phantom test protocol for this purpose. However, the ACR protocol recommends manual measurements, which are time‐consuming, labor‐intensive, and prone to variability, impacting accuracy and reproducibility. Although some aspects of the ACR evaluation have been automated or semi‐automated, tests like low‐contrast object detectability (LCOD), remain challenging to automate. LCOD involves assessing the visibility of objects at various contrast levels.

**Purpose:**

The purpose of this research is to propose and evaluate an automated approach for LCOD testing in MRI.

**Methods:**

The automated Python code generates a one‐dimensional profile of image intensities along radial paths from the center of the contrast disk. These profiles are compared to templates created from the disc's geometric information using general linear model statistical tests. A total of 80 image volumes (40 T1‐ and 40 T2‐weighted) were assessed twice by two human evaluators and the proposed Python code.

**Results:**

Human raters showed intra‐rater variability (Cohen's Kappa 0.941, 0.962), while the Python code exhibited perfect intra‐rater agreement. Inter‐rater agreement between the code and humans was comparable to human‐to‐human agreement (Cohen's Kappa 0.878 between the two human raters vs. 0.945, and 0.783 between the code and human raters). A stress test revealed both human raters and the code assigned higher scores to lower bandwidth images and lower scores to higher bandwidth images.

**Conclusion:**

The proposed automated method eliminates intra‐rater variability and achieves strong inter‐rater agreement with human raters. These findings suggest the method is reliable and suitable for clinical settings, showing high concordance with human assessments.

## 1 INTRODUCTION

Magnetic resonance imaging (MRI) is a crucial tool in clinical practice, requiring rigorous quality assurance (QA) procedures to ensure accurate patient diagnoses and treatment regimens.^1^, ^2^ QA protocols are essential in ensuring the stable performance of MRI systems and detecting any deviations in parameters that may impact image quality.^3^ Establishing early‐warning systems is essential for proactively detecting potential MRI scanner malfunctions.^3^, ^4^ The introduction of standardized MRI phantom test protocols by the American College of Radiology (ACR) in 2004 significantly enhanced the efficacy of QA procedures. These protocols utilize MRI phantoms, specifically designed objects with known compositions and geometries, to ensure the precise functionality of MRI instruments. The integration of large ACR phantom testing, along with the established MRI phantom test protocols, has established a robust framework for ensuring the reliability of MRI scanners around the world.^1^, ^2^

The ACR manual outlines the implementation of seven standardized tests: (1) geometric accuracy, (2) high‐contrast spatial resolution, (3) slice thickness accuracy, (4) slice position accuracy, (5) image intensity uniformity, (6) percent‐signal ghosting, and (7) low‐contrast object detectability (LCOD).^5^ The ACR instruction guides users through a series of manual steps to measure each test. While these procedures are straightforward, the inherent subjectivity and time‐intensive nature of the manual assessments have prompted the efforts to develop automated analytical methods, which offer improved accuracy, efficiency, and reproducibility.^1^, ^3^, ^6^, ^7^ Some of these tests are inherently quantitative and therefore are simpler to assess with automated codes. However, some of the tests, such as LCOD are inherently qualitative, and therefore present greater challenges in developing a code to automatically quantify them.

The evaluation of LCOD in MRI involves assessing the visibility of low‐contrast objects within an image.^5^ This assessment relies on the contrast‐to‐noise ratio to determine the detectability of the low‐contrast objects. This test is performed on slices 8 through 11 of a large ACR phantom scan, utilizing four sheets of plastic positioned at the inferior end of the phantom. Each slice covers one plastic sheet and each plastic sheet contains 30 holes arranged in 10 spokes that radiate from the sheet's center. The diameters of the spokes range from 7 mm for spoke 1 to 1.5 mm for spoke 10, following a clockwise sequence. The varying depths of the holes in different slices create varying levels of contrast, resulting from the partial volume effect. The contrast levels between the background and the holes vary across slices, corresponding to the differing thicknesses with contrasts of 1.4%, 2.5%, 3.6%, and 5.1% for slices 8 through 11, respectively. During the assessment, each slice is individually scored, beginning with the spoke with the largest diameter. The rater can adjust the image window and level as needed to enhance the visibility of the contrast objects. A spoke is considered as pass if all three holes are visible. However, the subjective nature of the scoring process often leads to variability in the number of complete spokes identified for each slice, thereby introducing inconsistencies in the test results. These challenges highlight the importance of standardized and automated evaluation techniques to enhance the reliability and reproducibility of LCOD tests.

Despite the challenges of automating the LCOD test, several efforts have been made to develop automated measurement methods. Some approaches rely on image processing techniques such as image intensity analysis^19^ and edge detection,^20^ while others employ machine learning and deep learning approaches.^21^, ^22^ Although each of these methods has its strengths, they also present specific limitations, such as the need for training data, reliance on predefined parameters, sensitivity to image artifacts, and limited agreement with human rater scores.

The primary objective of this research is to develop and evaluate an automated approach for LCOD testing in MRI quality assessments. This study aims to thoroughly evaluate the efficacy and accuracy of a proposed automated methodology in comparison to the conventional manual procedure. The assessment involves two experienced human raters to validate the consistency and reproducibility of the automated method in relation to the manual approach. By developing an automated analysis method for the LCOD test, the goal is to eliminate intra‐observer variability, while maintaining inter‐observer variability comparable to that of the manual procedure.

## 2 METHODS

### 2.1 Materials

**FIGURE 1:**
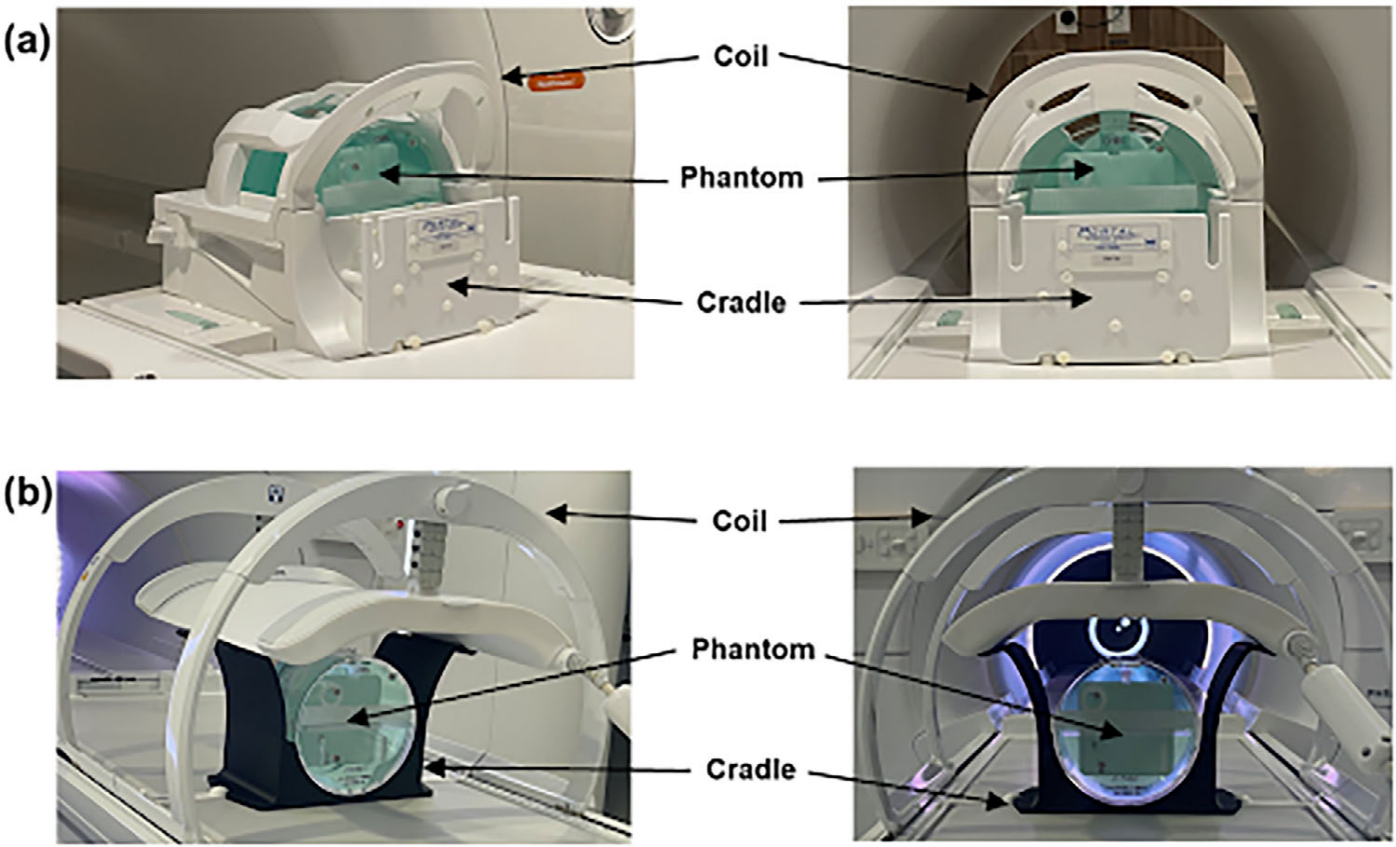
(a) Setup of 20‐channel head/neck coil with ACR phantom cradle and large ACR Phantom on 3.0T Siemens scanner. (b) Setup of eight‐channel coil with custom‐made cradle and large ACR Phantom on MR‐Linac system.

The images were acquired with the large ACR phantom using two MR scanners: a 3.0T Siemens MAGNETOM Vida MRI scanner (Siemens Healthineers, Erlangen, Germany) and a 1.5T Philips scanner integrated into an Elekta Unity MR‐Linac system (Elekta AB, Stockholm, Sweden; Philips Healthcare, Best, The Netherlands). A 20‐channel Head/Neck coil was used on the 3.0T Siemens scanner, while an 8‐channel coil (posterior 4 channel integrated into the table and anterior 4 channel positioned on top of the phantom using a coil bridge) was used on the MR‐Linac system (Figure 1). To ensure proper positioning of the large ACR phantom, an ACR Phantom Cradle (Portal Medical Group, North Logan, Utah, USA) was used with the 3.0T Siemens scanner, while a custom‐made cradle was employed for the MR‐Linac system.

### 2.2 Data acquisition

A total of 40 MRI datasets were analyzed in this study, with 20 acquired on the 3.0T Siemens scanner and 20 on the 1.5T MR‐Linac system. Each of these datasets contained two image volumes—one T1‐weighted and one T2‐weighted image volume, resulting in a total of 80 image volumes. Each image volume consisted of 11 slices of the large ACR phantom, slices number 8 to 11 of which were analyzed in this study. For the 3.0T Siemens scanner, 16 datasets were acquired using the standard acquisition parameters (Table 1). Four additional 3.0T datasets were collected during a single‐subject stress test, which included variations in imaging parameters: altered bandwidth (BW = 62 kHz and 781 Hz/Px), no pre‐scan normalization, and no distortion correction. For the 1.5T MR‐Linac system, 12 datasets were acquired using the standard protocol, and two more were obtained under standard conditions during stress test sessions. Two separate stress tests were also conducted on the 1.5T MR‐Linac system, each including four acquisitions: standard protocol, increased bandwidth (BW = 108 Hz/Px), no pre‐scan normalization, and no distortion correction—resulting in six altered‐parameter datasets (excluding the two regular acquisitions already counted). Whereas increasing the bandwidth to 781 Hz/Px was possible for the 3.0T scanner, it was not feasible for the 1.5T MR‐Linac system and therefore the bandwidth was only decreased to 108Hz/Px. All datasets, except for two additional 3.0T Siemens scanner stress datasets collected during the revision phase (used solely for stress testing), were included in intra‐ and inter‐rater agreement analyses. These modifications aimed to assess whether both the manual and automated methods could reliably identify changes in the images.

**TABLE 1:** Large ACR phantom scan parameters.

	3.0T		1.5T	
	T1‐weighted	T2‐weighted	T1‐weighted	T2‐weighted
Slices	11	11	11	11
FOV	250 × 250	250 × 250	250 × 250	250 × 250
Bandwidth (Hz/Px)	260	260	419	419
Slice thickness (mm)	5.0	5.0	5.0	5.0
TR (ms)	500.0	2000.0	500.0	2000.0
TE (ms)	20.0	80.0	20.0	80.0
Flip angle (°)	90	90	90	90
Resolution	256 × 256	256 × 256	256 × 256	256 × 256

Each of the 80 image volumes were independently and blindly assessed twice by each of the two human evaluators and the proposed Python code. Pass‐fail scores were assigned for the 10 spokes in each of the slices resulting in a total of 40 spokes evaluated per dataset. The ACR guidelines were followed for the manual scoring process, which was conducted using ImageJ software.^8^

### 2.3 Automated method

**FIGURE 2:**
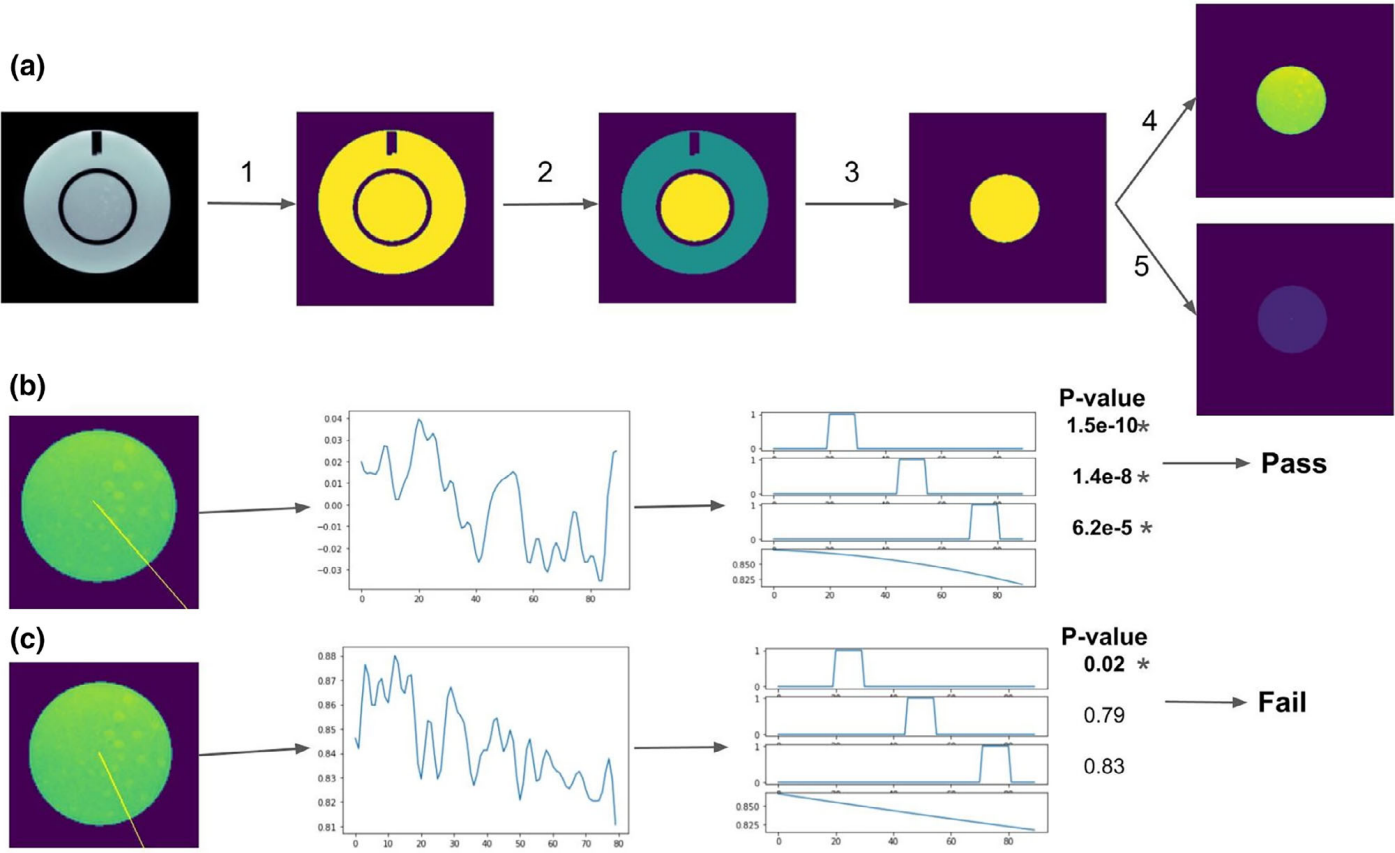
Visualization of the proposed algorithm for low contrast object detectability test: (a) (1) Image is binarized using histogram thresholding to remove background, (2) Connected components are identified and labeled, (3) A binary mask of the inner disk is generated by thresholding the labeled image, (4) The mask is used to remove outer disk, and (5) Center of gravity (COG) of the binary mask is calculated, which represents center of the disk. From the COG, a radial profile is generated at a designated angle, and the image intensity along this profile is sampled into a 1D array. The 1D profile is then matched against a predefined 1D template, which is created based on geometric information of the discs. (b) An example of a passed profile, where all three discs have profiles significantly different from background. (c) An example of a failed profile.

We developed an automated Python code based on statistical tests to determine whether the image intensity of the contrast holes differed significantly from the background (Figure 2). The code begins by normalizing the image intensity of each slice within a range of 0 to 1. Next, a background removal process is performed using histogram thresholding to isolate the relevant regions. Then the contrast disk is identified by detecting and labeling connected components in the binary image. This process results in a binary mask that outlines the inner circle containing the contrast objects.

The center of the circle is then identified by calculating the center of gravity (COG) of the binary mask. Using the COG as a reference, an angular radial profile in a specific angle is generated, sampling the image intensity along the profile into a 1D array. To ensure the 1D profile remains within the contrast disk, the profile is truncated at the disk's edge where the intensity drops to zero. For consistency across various imaging parameters, the 1D profile is resampled to include 90 samples, approximately equivalent to a resolution of 0.5mm. The angles of the first spoke in each slice is predefined based on the phantom's geometry, under the assumption that the phantom is perfectly positioned. However, the code generates several spokes within a range of ± 8 degrees around this initial angle.  This helps the code to compensate for slight phantom rotation.

The code incorporates the known structure of the contrast circles to generate expected 1D templates. The contrast objects, ranging from 7 to 1.5 mm in a clockwise progression, are positioned with the centers of the circles at distances of 12.5, 25, and 38 mm from the COG of the contrast disk. Leveraging the geometrical attributes of the contrast objects, individual 1D templates are created for each spoke. To account for potential intensity non‐uniformities, a second‐order polynomial is fitted to the profile and added into the general linear model (GLM) regressors. According to the ACR manual, a spoke is considered to pass if all three disks are identifiable. Therefore, we created three regressors for each 1D profile, one for each of the three disks. A test passes when all three GLM regressors exceed the significance level, ensuring the detectability of all three circles within a spoke. Considering there are four slices of low‐contrast objects, the significance level for each slice is set to 0.0125 (0.05 divided by 4, Bonferroni correction across the four slices). To control for multiple comparisons, the significance within each slice is adjusted using the Benjamini‐Hochberg false discovery rate.^9^ If all three objects in a spoke pass the corrected significance level, that spoke is considered to have passed the test. The number of passing spokes is then summed across all four slices.

### 2.4 Statistical tests

R Studio (Posit Software, PBC, version 2023.03.0) was used to perform statistical tests. Krippendorff's alpha^10^ and Cohen's Kappa^11^ with squared weighting were calculated to assess intra‐rater and inter‐rater agreement. For inter‐rater agreement, the two scores of each rater were averaged; whereas for intra‐rater agreement the datasets were kept separate and tested for both raters and the Python code. Moreover, the intraclass correlation coefficient (ICC)^12^ was calculated using a one‐way random effect model to measure the overall agreement among the three raters. Furthermore, Bland‐Altman Plots^13^ were generated to visually demonstrate inter‐rater and intra‐rater agreement.

To assess whether the code behaves similarly to human raters in response to changes in the images, a stress test was performed on 10 sets of image volumes (5 T1‐ and 5 T2‐weighted) with altered imaging parameters. For images acquired on the 3T Siemens scanner, each image volume set included five versions: regular, no pre‐scan normalization, no distortion correction, low bandwidth, and high bandwidth, whereas image volume sets from the 1.5T Philips scanner included four versions: regular, no pre‐scan normalization, no distortion correction, and low bandwidth. This involved a two‐way ANOVA test with Image Type and Rater as factors. The absence of significant interaction between the Rater and Image Type indicates consistent behavior between the rates and the code.

The LCOD test is sensitive to the image signal to noise ratio (SNR). We assessed the effect of SNR on the LCOD scores by calculating the correlation between SNR and the detectability score for each rater. Slice 7 from each scan is used to calculate SNR, which is measured as the ratio of the mean signal intensity within a region of interest in the central portion of the phantom to the standard deviation of the background noise.

## 3 RESULTS

Cohen's Kappa and Krippendorff's alpha were calculated to assess intra‐rater and inter‐rater agreement, with squared weighting used for the calculations. The results for Cohen's Kappa and Krippendorff's alpha are presented in Table 2.

**TABLE 2:** Cohen's Kappa and Krippendorff's alpha results for inter‐rater versus intra‐rater agreement.

	Inter‐rater agreement			Intra‐rater agreement		
	Rater 1 vs. Rater 2	Rater 1 vs. Python Code	Rater 2 vs. Python Code	Rater 1	Rater 2	Python Code
Kappa	0.878	0.783	0.945	0.941	0.962	1
Z	8.32	7.84	8.5	8.42	8.61	5.66
*p*	0	4.4e‐15	0	0	0	1.54e‐08
Krippendorff Alpha	0.872	0.792	0.875	0.885	0.948	1

As expected, the Python code demonstrated perfect intra‐rater agreement (1), while raters and the code demonstrated substantial inter‐rater agreement. On the other hand, manual raters exhibited substantial to almost perfect intra‐rater agreement (0.941 and 0.962) and substantial inter‐rater agreement (0.878).^14^ These results were found to be statistically significant as all *p* values were less than 0.05.

The ICC was also calculated using the averages of the two datasets, resulting in an ICC of 0.875 with F(79,160) equal to 22.1, and *p* < 0.001. The 95% confidence interval for the ICC population values was found to be 0.826 < ICC < 0.914.

**FIGURE 3:**
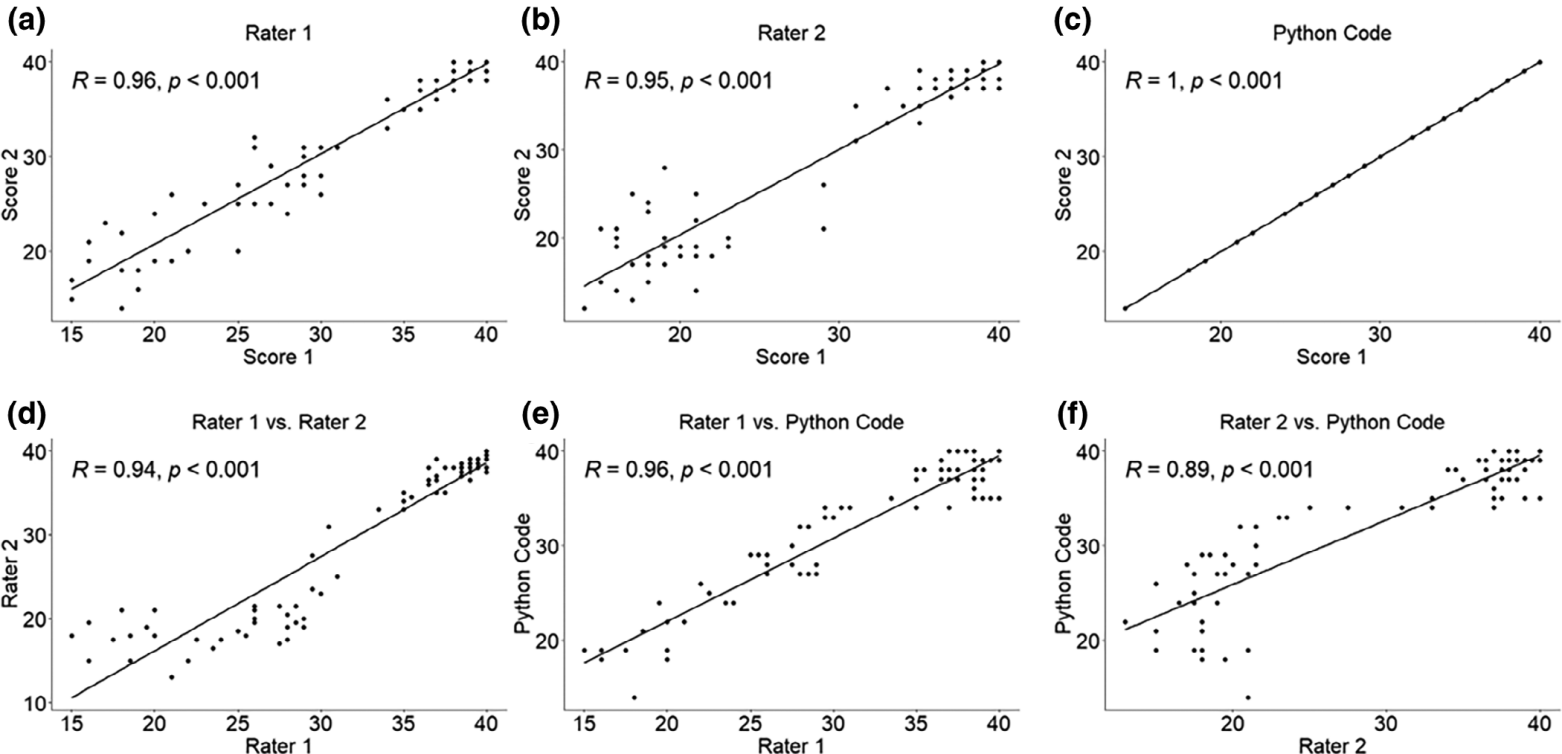
Correlation plots for intra‐rater agreement. (a) Rater 1, (b) Rater 2, and (c) Python code, as well as correlation plots for inter‐rater agreement (d) Rater 1 vs. Rater 2, (e) Rater 1 vs. Python code, and (f) Rater 2 vs. Python code. For each correlation plot, the correlation value and its corresponding p‐value is presented on the top left corner of the plot.

Figure 3 demonstrates correlations plots. The top row represents intra‐rater correlations between the two scores of each rater, while the bottom row represents inter‐rater correlations between the average scores of each pair of raters. The intra‐rater scores of the two human raters demonstrated substantial correlation (0.95 and 0.96), whereas the code produced perfect correlation, indicating the removal of intra‐rater variability. Moreover, the correlation between the human and code scores was comparable to the correlation between the scores of the two human raters.

**FIGURE 4:**
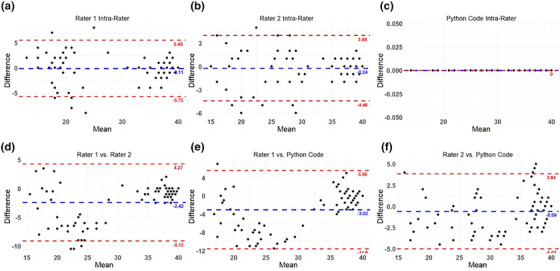
Bland‐Altman plots for intra‐rater agreement. (a) Rater 1, (b) Rater 2, (c) Python code, as well as Bland‐Altman plots for inter‐rater agreement (d) Rater 1 vs. Rater 2, (e) Rater 1 vs. Python code, and (f) Rater 2 vs. Python code. For each Bland‐Altman Plot, the dashed red lines correspond to the mean of the difference ± 1.96 standard deviation of the difference and the dashed blue line corresponds to the mean of the difference alone.

Bland‐Altman Plots for inter‐rater agreement and intra‐rater agreement are presented in Figure 4. Overall, no systematic differences or bias was detected between the Python code and human raters.

**FIGURE 5:**
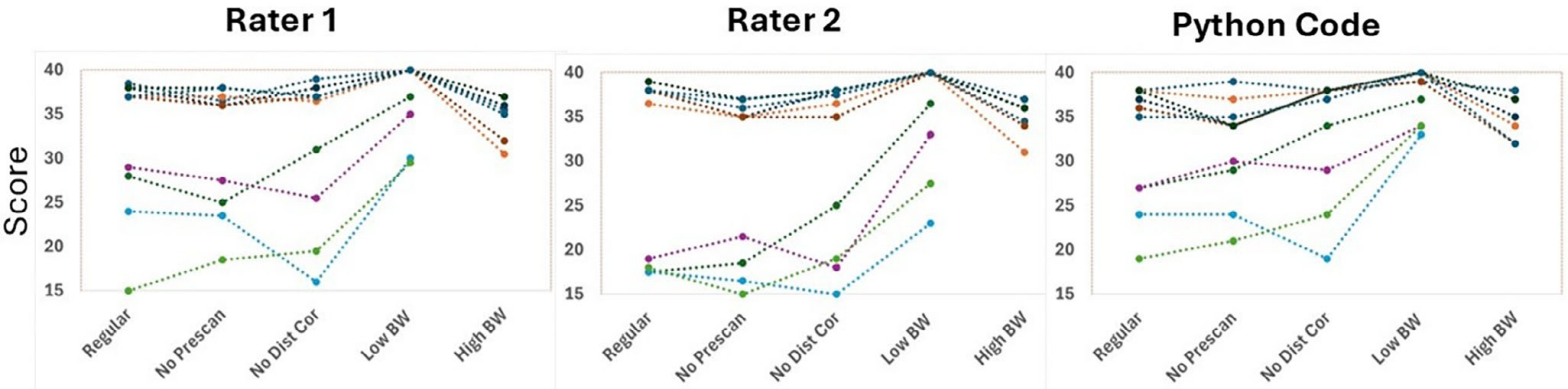
Results of the stress test for 10 image volume sets (6 from the 3.0T Siemens scanner and 4 from the 1.5T MR‐Linac system), showing the performance of the two human raters and the Python code. Images with lower bandwidth consistently received higher scores, and images with higher bandwidth received lower scores from both the human raters and the Python code, compared to other image types.

A stress test for 10 sets of image volumes was also performed (Figure 5). Results of the two‐way ANOVA test demonstrated a significant effect of Image Type (F = 4.07, *p* < 0.004), with no significant effect of Rater (F = 1.18, *p* < 0.31) or the Image Type x Rater interaction (F = 0.10, *p* < 0.99). This suggests no difference between the Python code and the two raters in scoring images with different parameters. A follow‐up *t*‐test indicated that images with lower bandwidth consistently generated higher scores compared to the other three image types, whereas higher bandwidth corresponds to lower scores across all raters.

**FIGURE 6:**
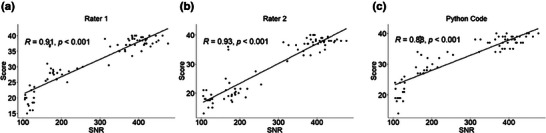
Correlation between the image SNR and the LCOD scores for (a) Rater 1, (b) Rater 2, and (c) Python code.

The association between the SNR values and low contrast detectability is assessed for each rater. The SNR values ranged from 100 to 450. The results, shown in Figure 6, reveal a strong and significant correlation between image SNR and low contrast detectability score for both human raters and the Python code. This indicates that the code exhibits behaviors consistent with that of human raters. Using a 3T scanner with a 20‐channel head and neck coil and a 1.5T scanner with an eight‐channel coil, we acquired images spanning a wide range of SNR. The python code performed reliably across this SNR range, demonstrating its robustness to SNR variability.

## 4 DISCUSSION

In this study, we presented a Python code that fully automates the LCOD test for large ACR phantom data. We validated the code using images from two MRI scanner manufacturers, two field strengths, and two receiver coils. All automatically calculated values were cross‐checked with manual image scoring to ensure the functionality of the proposed approach. Manual QC results from two human evaluators were used to assess within‐ and between‐rater variabilities. The proposed automated method eliminated intra‐rater variability and demonstrated acceptable inter‐rater agreement when compared with the human raters. The ICC also demonstrated moderate reliability of the testing methods.^15^ The results revealed that the automatically scored values were in good agreement with the manually acquired values.

Moreover, we conducted a stress test to evaluate whether the code exhibits the same sensitivity to changes in image quality (due to variations in imaging parameters) as the human raters. In the stress analysis, both the human raters and the Python code scored images with lower bandwidth significantly higher and images with higher bandwidth significantly lower. This outcome is aligned with expectations, as reducing bandwidth decreases image noise, thereby enhancing the detectability of contrast objects.^16^ Changes to other imaging parameters did not affect the LCOD scores for either the human raters or the code.

Disabling the pre‐scan normalization filter generates less uniform images.^17^ However, in the manual scoring process, human raters adjust the display window and level to counteract the effect of nonuniformity. Therefore, the pre‐scan normalization filter is not expected to change the LCOD score. As anticipated, this alteration did not affect the scores assigned by the two human raters. Similarly, the code mirrored this behavior and assigned comparable scores to images regardless of pre‐scan normalization.

Disabling gradient nonlinearity correction introduces distortion into the image.^18^ However, LCOD scores are not sensitive to geometric distortion. As expected, this change did not significantly affect the scores assigned by the two human raters. While the code relies on geometry information of the contrast objects, the stress test demonstrated that it is robust against the disabling gradient nonlinearity correction. This robustness is likely due to the minimal distortion within the small field of view associated with the ACR phantom size. In addition, the 1D profiles were normalized by resampling, which helps reduce the impact of distortion‐related changes in profile length due to image distortion. To further mitigate the effects of image distortion, the code also allows for the slight jittering of the 1D profiles—up to five samples—to better align them with the templates. These small profile adjustments implemented in the code helped mitigate the effect of image distortions. Overall, the stress test demonstrated that the code was able to reliably identify changes in the image quality that impact the score of the LCOD test.

Although the initial spoke angles are determined based on the phantom's geometry and position, the code searches around these angles to identify spokes that meet a significant threshold. Therefore, the code is expected to be insensitive to phantom rotations. To test this, we rotated the phantom from ‐10 to +10 degrees in 2.5 degrees increments. The results (not shown) indicated that rotations up to 5 degrees did not significantly change the scores. However, rotating beyond 5 degrees resulted in failures in detecting spokes corresponding to smaller discs.

Traditionally, the assessment of LCOD has been performed manually, mirroring the way clinical images are evaluated by radiologists and other healthcare professionals. As a result, the LCOD test has depended entirely on the visual perception of an experienced operator, making it observer‐ and monitor‐dependent.^7^ However, with the ongoing advancements in image processing techniques within the clinical context, medical images should be interpretable not only by humans but also by computer systems. Using a computer program for a QA test can provide more consistent, objective, and reproducible results, reducing human error and improving the overall efficiency of the process.

Other researchers have also explored efforts to automate the LCOD test. Fitzpatrick et al.^19^ utilized the correlation between radial profiles and expected templates to identify detectable spokes. However, defining a subjective correlation threshold is challenging, and interpreting the correlation itself can be complex. For example, a radial profile might show a high correlation with the template even if only two out of three disks are detectable. In another approach, Ehman et al.^20^ developed a code that utilizes edge detection and other image processing techniques to automatically score LCOD images. Their method achieved inter‐rater variability comparable to that of human raters. However, their algorithm is computationally intensive and involves multiple parameters, the configuration of which is neither straightforward nor intuitive—such as the use of different criteria and thresholds for various spokes and slices. Moreover, because the algorithm relies heavily on edge detection, factors such as image noise, Gibbs artifacts, and other distortions can significantly impair its performance. Ramos et al. developed two methods, one based on machine learning^21^ and another based on deep learning,^22^ achieving results comparable to those of experienced raters. However, since the models are trained on data scored by human raters, their performance is heavily dependent on the quality of the training data. Although users can customize the model using their own data, this customization may limit the algorithm's generalizability.

While each of the previously developed code algorithms have their own strengths, our model offers several distinct advantages. It is entirely objective, does not require a training dataset, and is straightforward to understand, relying solely on statistical tests. To maintain its objectivity, we did not adjust the significance threshold in our statistical tests to align with human rater scores. Despite this, our method demonstrated stronger agreement with experienced human raters compared to previous studies—Ehman et al. reported a Krippendorff's alpha of 0.652, while our study achieved values of 0.792 and 0.875. Moreover, users have the flexibility to adjust the significance threshold to obtain either more conservative or more relaxed results, depending on their specific needs.

## 5 CONCLUSION

This study introduced and validated an automated approach for LCOD testing, addressing a critical gap in MRI QA. The method demonstrated perfect intra‐rater reliability, substantial inter‐rater agreement comparable to human evaluators, and high sensitivity to imaging parameter changes, such as bandwidth adjustments. These results underscore its reliability and precision in assessing LCOD performance.

The Python code eliminates the subjectivity and variability of manual LCOD assessments by providing objective, reproducible, and efficient evaluations. Its reliance on straightforward statistical modeling avoids the complexity of machine learning approaches, making it adaptable and easy to implement across diverse clinical and research environments.

In conclusion, this automated LCOD testing method advances MRI QA by improving consistency and efficiency while maintaining sensitivity to imaging quality. Its broad applicability and simplicity make it a valuable tool for long‐term performance monitoring and enhancing diagnostic reliability. Future work may explore its integration into comprehensive QA protocols and additional imaging applications. Moreover, similar Python codes will be developed for medium and small ACR phantom.

## AUTHOR CONTRIBUTIONS

Data acquisition: Ali M. Golestani; Python code programming: Ali M. Golestani; Manual method measurements: Ali M. Golestani and Julia M. Gee.; Data analysis: Ali M. Golestani and Julia M. Gee.; Literature review, manuscript draft and editing: Ali M. Golestani and Julia M. Gee Both authors have read and agreed to the published version of the manuscript.

## CONFLICT OF INTEREST STATEMENT

The authors declare no conflict of interest.

## DATA AVAILABILITY STATMENT

The data that support the findings of this study are available from the corresponding author upon reasonable request. The Python code is available in the following repository: https://github.com/aligoles/ACR‐Low‐Contrast‐Object‐Detectability
